# Epidemiological Patterns of Animal Bites in Babol City, Northern Iran (2015-2022)

**DOI:** 10.34172/aim.34613

**Published:** 2025-08-01

**Authors:** Seyedeh-Zahra Hosseini-Larijani, Mohsen Karami, Mohammad-Reza Adel-Mashhadsari, Rahim Malekzadeh-Kebria, Hassan Heydari, Hossein-Ali Nikbakht

**Affiliations:** ^1^Student Research Committee, Babol University of Medical Science, Babol, Iran; ^2^Infectious Diseases and Tropical Medicine Research Center, Health Research Institute, Department of Parasitology and Mycology, Babol University of Medical Sciences, Babol, Iran; ^3^Department of Health, Health Systems Research, Health Research Institute, Babol University of Medical Sciences, Babol, Iran; ^4^Social Determinants of Health Research Center, Health Research Institute, Babol University of Medical Sciences, Babol, Iran

**Keywords:** Animal bites, Epidemiology, Injury, Rabies

## Abstract

**Background:**

Diseases resulting from animal bites have been a public health concern globally, including in Iran, and continue to show annual growth. Therefore, this study aimed to investigate the epidemiological patterns of animal bites in Babol city over 8 years.

**Methods:**

This cross-sectional study was conducted on all individuals injured by animal bites from March 2015 to February 2022. The data for these cases were recorded by trained professionals at the Central Rabies Center in the county.

**Results:**

A total of 13,550 cases with a mean age of 36.18±35.86 years were examined. Three-quarters of the cases (76.0%) were male, and 48.7% were urban residents. Additionally, 11,504 individuals (84.9%) received three doses of the rabies vaccine. The trend of animal bites showed an increase from 199.07 cases per 100,000 population in 2015 to 547.98 cases per 100,000 population in the final year of the study (2022) (*P*<0.001). This increasing trend was also observed in both males (from 327.03 in 2015 to 783.36 in 2022) and females (from 69.35 in 2015 to 309.36 in 2022). In terms of gender, the prevalence of animal bites in males was 1.3 times higher than females, and the prevalence of animal bites in rural areas was 59% higher than urban areas.

**Conclusion:**

The occurrence of animal bites showed an increasing trend during the study years, indicating growing concern and the need for effective preventive measures. Furthermore, the results can assist in developing strategies, policies, and public health interventions in Babol.

## Introduction

 Animal bites, resulting from the bite or scratch of a domestic or wild animal, can lead to significant morbidity and mortality, especially in rural areas, and are an important public health issue that has been neglected.^1-3^ Rabies is the deadliest disease transmitted through animal bites. This disease is one of the most dangerous viral zoonoses, and all mammals can be infected. The causative agent of this disease is a neurotropic virus belonging to the Rhabdoviridae family and the Lyssavirus genus.^4^ These diseases are transmitted to humans through inhalation, scratching, licking of mucous membranes, or broken skin that comes into direct contact with the saliva of infected animals.^5^ Among these, approximately 85% to 90% of animal bite injuries occur by dogs, 5% to 10% by cats, and 2% to 3% by humans and rodents.^6^ In addition to the issue of human health, the occurrence of the disease in animals causes significant economic losses.^7^

 According to a study by the World Health Organization, more than 3 billion people in Asian countries have been exposed to rabies, with over 30,000 deaths annually due to rabies in these countries. In other words, one Asian dies every 15 minutes due to rabies, and importantly, 15% of these deaths are recorded in children under 15 years of age.^8^

 Iran is among the endemic countries for rabies, and all provinces of Iran are more or less affected by this disease. The spread of this disease is not unexpected given the lack of disease control in domestic and wild animals.^2,5,7,9^ According to the results of a meta-analysis, the incidence of animal bites in Iran is 13.2 cases per 1000 people, with this number being three times higher in males than females. Additionally, in this study, the incidence of animal bites in rural areas was 17.45 per 1000 people compared to 4.35 per 1000 people in urban areas. The incidence of animal bites in domestic animals is higher than wild animals, with dogs having the highest incidence rate.^10^

 There is no effective treatment for rabies, and only a few patients have survived intensive care in specialized care units.^11^ However, rabies is preventable through vaccination, and most deaths from rabies are due to lack of awareness and poor access to healthcare services.^12,13^ Prevention is indeed the only way to treat rabies, which includes vaccinating domestic animals, raising awareness, and monitoring. The increasing population of stray dogs and the expanding cases of animal bites and rabies in many provinces of the country emphasize the need for greater attention to disease control and research in various aspects of rabies.

 Despite significant advances in prevention and treatment, various studies still indicate an increase in annual cases of rabies due to increased public awareness leading to more referrals, increased ownership of domestic animals, especially in urban areas, and the rise in the number of stray dogs in recent years. This increase, in addition to causing fatalities, will lead to increased costs in the health sector for prevention programs and vaccination. On the other hand, to prevent rabies, accurate information is needed on the epidemiological patterns of animal bites to implement appropriate health interventions. Moreover, the wide geographical range, climatic diversity, and demographic differences in terms of health and awareness necessitate separate investigations in different regions of the country. Therefore, this study was conducted to examine the epidemiological patterns of animal bites in the Babol County during the years 2015-2022.

## Materials and Methods

 In this cross-sectional study, we examined the individual, environmental, temporal, and animal-related patterns contributing to animal bite incidents in Babol city over 8 years (from March 2015 to February 2022).

 This study used a census method, including all registered cases of animal bites recorded in the official Ministry of Health database between 2015 and 2022 in Babol. As the data were extracted from a comprehensive registry, no sampling was performed, and the entire population of recorded cases were analyzed.

 In this study, we included all individuals injured by animal bites in Babol, whose information was recorded in the county’s rabies center files by trained experts, and registered in the Ministry of Health of Iran’s animal bite registration system according to relevant guidelines. Eventually, a total of 13,550 cases participated in the study. This research was approved by the Ethics Committee of Babol University of Medical Sciences under the ethics code (MUBABOL.HRI.REC.1402.141). Permission was also obtained from the health center of Babol.

 A checklist was prepared based on the information recorded in the animal bite registration offices at the Babol Health Center (Infectious Diseases Unit - Rabies Department), where the data on these animal bite incidents was entered into the Ministry of Health’s Infectious Diseases Management System. The data entered into this software system included personal variables such as age, gender, occupation, location of injury, time of injury, day of injury, month of injury, type of injury, type of animal (dog, cat, etc.), injured body part, type of injury, wound size, delay time, received rabies serum, vaccination, previous vaccination history, number of vaccination doses, tetanus vaccine, time pattern (day, season, year of the incident), and type of biting animal (domestic/wild), as well as the animal’s status after ten days. Additionally, to calculate the incidence rate of animal bites, population estimates were made for the years under study. For this purpose, population estimates were calculated for the relevant years, both overall and disaggregated by age groups, by considering the population growth over the census years.

 In the data analysis process, mean and standard deviation were reported for quantitative variables and frequency and percentage were reported for qualitative variables to show descriptive statistics. Analytical statistics were conducted using the Chi-square test. The assumptions of the Chi-square test were evaluated and met; no expected cell count was below 1, and less than 20% were below 5. Given the descriptive epidemiological design of this study, the majority of findings are presented using descriptive statistics. Where applicable, association measures such as odds ratios with 95% confidence intervals were reported to assess the magnitude and direction of effects. Additionally, the trend of animal bite cases over the mentioned years was analyzed using the Cochran-Armitage trend test. All analyses were performed using SPSS version 23, and Microsoft Excel 2013 was utilized for drawing graphs. The significance level was considered at *P* < 0.05.

## Results

###  Demographic Characteristics

 During the 8-year study period, data on 13,550 cases of animal bites in Babol city between 2015 and 2022 were collected. The results showed that the majority of cases (27.9%) were in the age group of 31-45 years, with a mean age and standard deviation of 35.86 ± 18.36 years. Three-quarters of the cases (76.0%) were male, 48.7% were urban residents, and only 0.2% were non-Iranian nationals. Regarding occupation, the majority (43.3%) were self-employed, followed by 17.8% students, and 14.0% housewives. The demographic characteristics of the study participants are presented in Table 1.

**Table 1:** Investigating the Relationship Between Delay in Treatment and Demographic Characteristics and Bite Details of Animal Bite Cases in Babol During 2015-2022

**Variable**		**Frequency (present)**	**Initial treatment time**		***P*** value^*^
			**≤48 hours** **N (%)** **13398 (98.9)**	**>48 hours** **N (%)** **152 (1.1)**	
Age (y)	< 7	504 (3.7)	493 (97.8)	11 (2.2)	0.273
	7-18	2071 (15.3)	2050 (99.0)	21 (1.0)	
	19-30	3131 (23.1)	3093 (98.8)	38 (1.2)	
	31-45	3780 (27.9)	3738 (98.9)	42 (1.1)	
	46-60	2644 (19.5)	2617 (99.0)	27 (1.0)	
	> 60	1420 (10.5)	1407 (99.1)	13 (0.9)	
Gender	Male	10303 (76.0)	10191 (98.9)	112 (1.1)	0.494
	Female	3247 (24.0)	3207 (98.8)	40 (1.2)	
Residential area	Urban	6597 (48.7)	6526 (98.9)	71 (1.1)	0.624
	Rural	6953 (51.3)	6872 (98.8)	81 (1.2)	
Season of the bites	Spring	3595 (26.5)	3554 (98.9)	41 (1.1)	0.191
	Summer	3533 (26.1)	3504 (99.2)	29 (0.8)	
	Fall	3202 (23.6)	3164 (98.8)	38 (1.2)	
	Winter	3220 (23.8)	3176 (98.6)	44 (1.4)	
Time of the bites (h)	0-6	845 (6.2)	837 (99.1)	8 (0.9)	0.969
	6-12	36.0 (26.6)	3561 (98.9)	41 (1.1)	
	12-18	4597 (33.9)	4545 (98.9)	52 (1.1)	
	18-24	4506 (33.3)	4455 (98.9)	51 (1.1)	
Type of animal	Dog	11155 (82.3)	11045 (99.0)	110 (1.0)	0.001
	Cat	1615 (11.9)	1600 (99.1)	15 (0.9)	
	Mouse	536 (4.0)	530 (98.9)	6 (1.1)	
	Other	244 (1.8)	223 (91.4)	21 (8.6)	
Domestic	No	9080 (67.0)	9003 (99.2)	77 (0.8)	0.001
	Yes	4470 (33.0)	4395 (98.3)	75 (1.7)	

^*^Chi- Square test.

###  Bite Characteristics

 In terms of the season of the bites, approximately one-fourth of the cases occurred in each season. Of the total number of cases, 4597 (33.9%) occurred between 12:00 to 18:00, and 4506 (33.3%) occurred between 18:00 to 24:00. The majority of bites were by dogs, accounting for approximately 11,155 (82%), followed by cats and rodents at 1615 (11.9%) and 536 (4%), respectively (Table 1). Additionally, out of all the animals involved, 4470 (33%) were domestic, and about half of the animal bite victims sustained one injury. Furthermore, 7255 (53.5%) individuals were bitten suddenly, and in 4956 (36.6%) cases, the victims were the owners of the animals. Over 8 years, eight (0.1%) cases resulted in death.

###  Vaccination Status

 Regarding the number of rabies vaccine injections, 11,504 individuals (84.9%) received three doses of the vaccine, and 13,398 (98.9%) sought zero-dose vaccination within 48 hours after the animal bite. Additionally, 11 cases of adverse reactions to the zero-dose vaccine were reported among all individuals. Furthermore, out of all cases, only 254 (1.9%) had a history of receiving the rabies vaccine alone, while 13,285 (98%) received a combined or tetanus vaccine.

###  Measures Taken in Animal Bite Treatment

 Regarding the measures taken in healthcare centers, 13,199 individuals (97.4%) underwent washing with soap and water, and disinfection was performed for 1,035 cases (7.6%). Only four individuals received wound dressing, and none of the cases required stitches. Moreover, antibiotics were prescribed for 308 cases (2.3%), and none of the individuals developed infections at the site of the injury.

###  Relationship Between Time of Seeking Zero-Dose Vaccination and Bite Characteristics

 The relationship between the time of seeking zero-dose vaccination and bite characteristics is presented in Table 1. The results indicate a statistically significant relationship (*P* < 0.05) between the type of animal and domesticity with the time of seeking zero-dose vaccination. Individuals bitten by dogs, cats, and mice tended to seek treatment within 48 hours more quickly compared to other bitten animals.

###  Odds Ratio for Chance of Animal Bite Incidence by Key Demographic Variables

 The odds ratio for variables of gender, residential area, and age group of bitten individuals is presented in Table 2. The results indicate that in the age groups of 10-19, 40-49, 70-79, and over 80 years, the incidence of animal bites was 79%, 89%, 78%, and 23% higher, respectively, compared to the age group of 0-9 years. Additionally, in the age groups of 20-29, 30-39, 50-59, and 60-69 years, the incidence of animal bites was 2.20%, 2.06%, 2.11%, and 2.25% higher, respectively, compared to the age group of 0-9 years. Regarding the gender variable, the incidence of animal bites in males was 3.21 times higher than females, and the incidence of animal bites in rural areas was 59% higher than urban areas.

**Table 2:** Prevalence Odds Ratio for the Variables of Gender, Residential Area and Age Category of Animal Bite Cases in Babol (2015-2022)

**Variable**		**Odds ratio**	**95% Confidence interval**
Age (y)	0-9	Reference	—
	10-19	1.79	1.65 – 1.94
	20-29	2.20	2.04 – 2.37
	30-39	2.06	1.92 – 2.22
	40-49	1.89	1.75 – 2.04
	50-59	2.11	1.98 – 2.28
	60-69	2.25	2.06 – 2.46
	70-79	1.78	1.59 – 2.00
	≥ 80	1.23	1.03 – 1.46
Gender	Female	Reference	—
	Male	3.24	3.08 – 3.24
Residential area	Urbon	Reference	—
	Rural	1.59	1.54 – 1.65

*Note:* The odds ratios presented are unadjusted and intended for descriptive purposes only, to illustrate crude associations with key demographic variables. No multivariable modeling was performed as the primary focus of the study is descriptive epidemiological surveillance.

###  Overall Trend of the Number and Rate of Animal Bites Incidence

**Figure 1:**
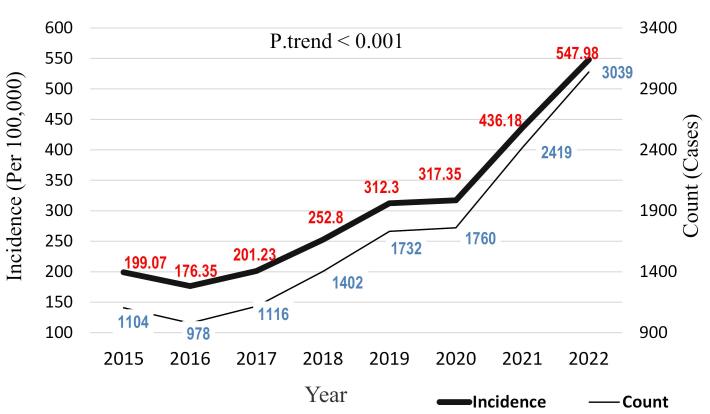
Trend of the Number and Incidence Rate of Animal Bites in 100,000 People of Babol (2015-2022)

 The incidence of animal bites during these years averaged 2443 per 100,000 thousand population of the city. The trend of animal bite incidence during the study years indicates an overall increasing trend. Although there were fluctuations over the years due to various reasons, the overall trend was increasing, such that in the initial year of the study in 2015, the incidence rate was 199.07 per 100,000 population, which increased to 547.98 per 100,000 population in the final year of the study (2022). The results of the Cochran-Armitage test showed that this increasing trend was statistically significant (*P *trend < 0.001) (Figure 1).

###  Trend of Animal Bite Incidence in Babol by Months of the Year

**Figure 2:**
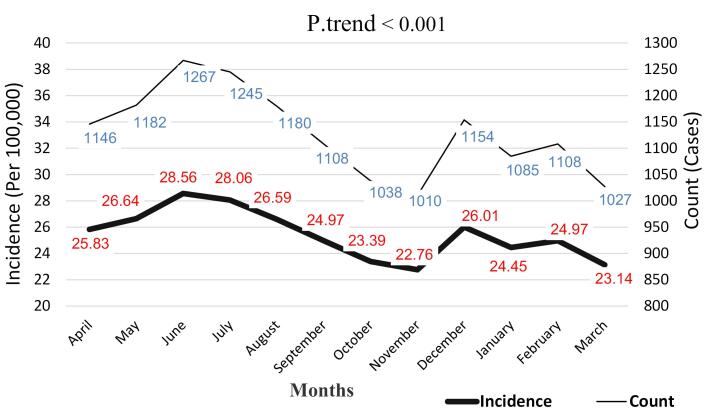
Trend of the Number and Incidence Rate of Animal Bites in 100,000 People of Babol During Different Months of the Year

 The highest number of animal bite cases, accounting for 9.4% (1267 cases) and an incidence rate of 28.56 per 100,000 population, occurred in May, while the lowest number was in October with 7.5% (1010 cases) and an incidence rate of 22.76 per 100,000 population. Analyzing the trend of animal bite occurrence during the study months reveals an overall decreasing trend. Although there were fluctuations throughout the months due to various reasons, the overall trend is decreasing, such that the incidence rate was 25.83 per 100,000 population in March, which decreased to 23.14 in February per 100,000 population (*P* trend < 0.001) (Figure 2).

###  Trend of Animal Bite Incidence by Gender

**Figure 3:**
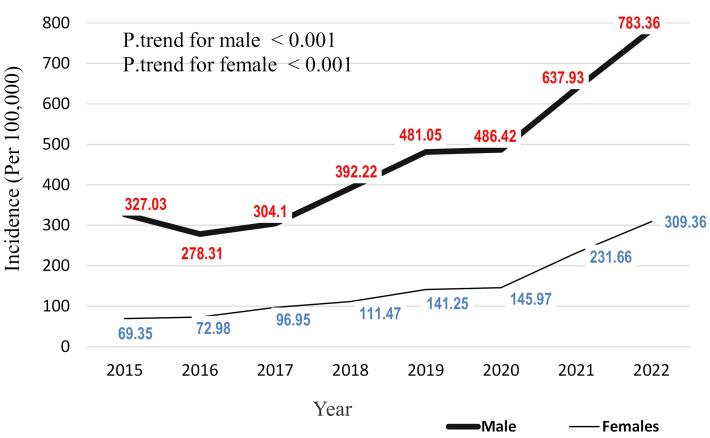
Trend of the Incidence of Animal Bites in 100,000 People of Babol by Gender (2015-2022)

 The incidence of animal bites over these years was not the same by gender. The overall incidence rate of animal bites in males and females was 3690.42 and 1178.99 per 100,000 population, respectively. Comparing the incidence of animal bites in the two groups of women and men during the study years shows that both genders experienced an increasing trend, which was statistically significant (P trend < 0.001). In 2015, the incidence rate of animal bites in men was 327.03, which increased to 783.36 per 100,000 population in 2022. Also, this trend in women started at 69.35 in the initial year of the study and increased to 309.36 per 100,000 population in the final year (Figure 3).

###  Trend of the Number and Rate of Animal Bites Incidence by Residential Area

**Figure 4:**
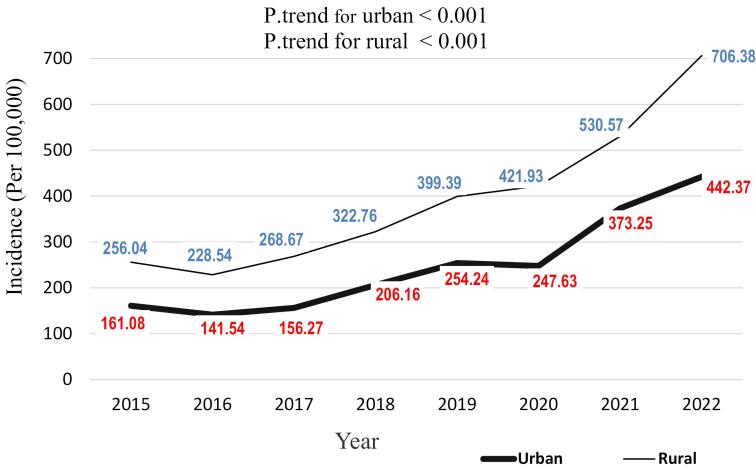
Trend of the Incidence of Animal Bites in 100,000 People of Babol by Place of Residence (2015-2022)

 The incidence rate varied by residential area, with the overall incidence rate of animal bites in urban and rural residents being 1982.56 and 3134.32 per 100,000 population, respectively. Animal bites showed an increasing trend in both urban and rural areas, with a slightly higher increase observed in rural residents. Both trends were statistically significant (P trend < 0.001). In 2015, the incidence rate in urban areas was 161.08, which increased to 442.37 per 100,000 population in 2022. Similarly, in rural areas, the incidence rate was 256.04 in the initial year, which increased to 706.38 per 100,000 population in the final year (Figure 4).

###  Trend of the Number and Rate of Animal Bites Incidence by Age Decades 

**Figure 5:**
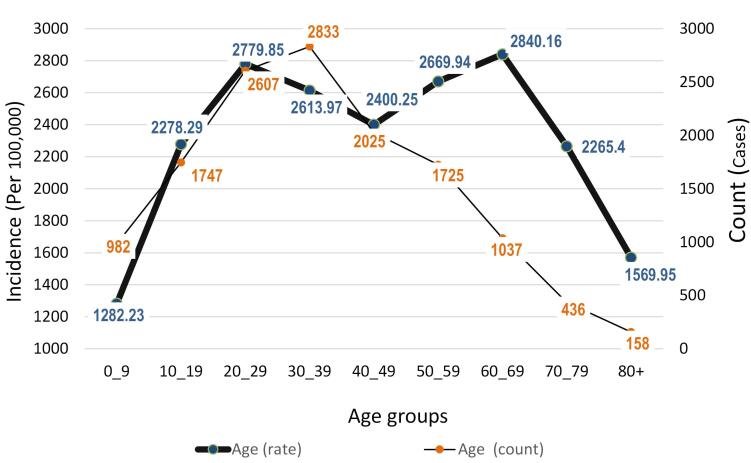
Trend of the Number and Incidence Rate of Animal Bites in 100,000 People of Babol by Age Decades (2015-2022)

 The highest number of cases, with 2833 individuals, was related to the age decade of 30-39 years, followed by the age decades of 20-29 and 40-49 years with 2607 and 2025 cases, respectively. The lowest number of cases was observed in the age group over 80 years. However, in terms of the incidence rate specific to age, the incidence of animal bites generally increased with each age decade. After the age decade of 60-69 years, there was a decreasing trend again. The lowest incidence rate was observed in the age group under 10 years with 1282.23 cases per 100,000 population, and the highest was in the age group of 60-69 years with 2840.16 cases per 100,000 population within the same age group (Figure 5).

## Discussion

 This study was conducted on 13,550 cases of animal bites to investigate the trend of animal bites and some associated risk factors in Babol city during 2015-2022. The results indicated an increasing trend in the incidence of animal bites in Babol. This increasing trend was analyzed both by gender and by place of residence. The results showed a relationship between age groups and gender in animal bite cases, with males having a higher percentage of bites in all age groups. The incidence of animal bites was higher in rural areas compared to urban areas, and the highest incidence was observed in males and the age group of 31-45 years. The majority of bites were attributed to dogs, accounting for approximately 11,155 cases (82%), followed by cats and rodents with 1615 (11%) and 536 (4%), respectively. Regarding the vaccinations, the majority of individuals received three doses of rabies vaccine, and about 99% sought post-exposure zero-dose vaccination within 48 hours of the bite.

 The eight-year trend of animal bite incidence in Babol city is increasing. Consistent with the findings of this study, a study by Amiri et al in 2020 aiming to examine the epidemiological patterns of animal bites during 2012-2019 concluded that the incidence of animal bites increased from 206.4 per 100,000 population in 2012 to 212.9 in 2019 and this trend was statistically significant ^13^. Another study in southern Iran from 2015 to 2019 reported an overall incidence of 142.93 per 100,000 population, showing a significant increasing trend.^14^ Additionally, a study conducted in Delhi in 2022 demonstrated an increasing trend in animal bite cases during the study years.^15^ Furthermore, studies conducted in various cities in Iran have indicated an increasing trend in animal bite cases from 2014 to 2018, and according to their charts, the trend is expected to continue to rise in the future.^16^ Other studies in Iran also report an increasing trend in animal bite cases.^17-19^

 Changes in weather patterns or other environmental factors, such as increased pet ownership in households, may influence animal behavior and distribution, potentially leading to increased human-animal interactions and subsequent bites. On the other hand, changes in public awareness or reporting practices over the years may also contribute to the observed trends. Increased awareness may result in more reporting of incidents, and improved reporting systems can provide more accurate data.

 In the present study, most cases of animal bites occurred in men and the age group of 31-45 years. The results indicated a relationship between age groups and gender in animal bite cases, with men having a higher percentage of bites in all age groups. A study in Khaf city, Khorasan Razavi province, showed that the majority of animal bite cases were in men (79.7%) and the age range of 21 to 60 years (48.5%).^20^ Another research in southern Iran in 2022 demonstrated a higher incidence of animal bites in men compared to women.^14^ In a study conducted by Aydin and colleagues in 2023, over 4 years, more cases of animal bites were reported in men than women (240 men and 179 women).^21^ A study in Kermanshah showed that 76.3% of the study population were men, and 34% were employed in non-governmental occupations.^18^ Another study in Khuzestan in 2018 reported that out of 2493 cases, 76.6% were men, and most bites occurred in the age group of 21 to 30 years.^22^

 A different study involving 1300 cases of animal bites found that 971 cases (74.7%) were men and 329 cases (25.3%) were women, and the highest incidence of bites (31.9%) was in the age group of 10 to 20 years.^19^ This finding regarding age groups does not align with the current study. Gender differences in animal bites may be related to different patterns of outdoor activities or exposure to environments where the likelihood of encountering animals is higher.

 In the present study, the incidence of animal bite cases was greater in rural areas compared to urban areas; in terms of occupation, the majority of cases were self-employed, students, and housekeepers. Comparisons showed that the incidence of animal bites is increasing both in rural and urban residents, but this increase is slightly higher in rural areas, and both are statistically significant. Ghaffari Fam’s research showed that 65.9% of animal bites occurred in rural areas.^23^ A study in Kermanshah indicated that 34% of individuals affected by animal bites were employed in non-governmental occupations,^18^ which is consistent with the findings of the current study.

 In line with the current research, a study by Kasiri et al in Khuzestan in 2018 showed that the majority of animal bite cases (24.7%) were self-employed, and overall, 65% of animal bite cases occurred in urban areas.^22^ Changes in population density, urbanization, or land use patterns may influence the frequency of interactions between humans and animals. For example, rural areas may experience more contact due to shared spaces between humans and animals.

 The results of this study indicated that the highest number of animal bites occurred in May and the lowest in October. In line with our study, a research in 2019 revealed a seasonal trend in the occurrence of animal bites. Consequently, the monthly number of animal bite cases gradually increased in the first month of each year, with the highest number of cases occurring from March to May, and then gradually decreasing until reaching its lowest level in October.^9^ Another study showed that animal bites are more common in spring (26.7%) and fall (25.2%). The highest number of animal bites was reported in March (9.2%) and May (9%) (23). This finding is consistent with the current study’s results regarding the higher incidence of animal bites in spring and summer. This may be due to seasonal changes, where environmental factors or animal behaviors in specific seasons can affect the likelihood of interactions between animals and humans.

 The results of the current study showed that most animal bites occurred between the hours of 12-18 and 18-24 and were attributed to dogs, cats, and rodents. Furthermore, in terms of the type of animal bites, men were more commonly bitten by dogs, while women were more commonly bitten by cats and rodents. Regarding the number of rabies vaccine injections, most cases received three doses of the vaccine, and they received zero-dose vaccination less than 48 hours after the bite. A study conducted in Najafabad also indicated an increase in animal bite cases by dogs, followed by cats.^13^ a study by Singh et al in 2023 demonstrated that rural areas have a high number of dog bites, with dogs being the most common biting animal.^24^ A retrospective and multicenter study by Yurtsever et al in 2023 showed that patients were mostly attacked by cats and dogs, and the majority of cases sought vaccination after animal bites.^25^ Aydin et al in 2023 showed that 51% were bitten by dogs and 47% by cats and 97% of patients received rabies vaccination.^21^ Other studies^18,19^ have also shown that, regarding the type of animal bite in all ages, dog damage was more than the damage caused by cats and other domestic or wild animals.

 This distribution may be influenced by the common presence of these pets in households. The high percentage of patients receiving the rabies vaccine indicates a preventive approach to potential rabies spread, highlighting the importance of timely medical intervention and awareness of the risk of infectious diseases. The higher occurrence of bites during specific hours may be because these periods coincide with increased human activity or interaction with animals. Dogs, cats, and rodents may be more active during these times, leading to a higher likelihood of bites. Men may be more exposed to dogs due to occupations or specific outdoor activities. On the other hand, women may spend more time in places where cats and rodents are common, such as homes or specific work environments.

 Investigating animal bite cases over 8 years, considering all relevant variables, and examining the trends and patterns of the disease are the strength points of this study. However, focusing on a specific geographical area may limit the generalizability of the findings to other areas with different populations and environmental factors. Additionally, the cross-sectional nature of the study and lack of investigation into potential background factors contributing to the observed trends, such as changes in animal behavior, population density, or socio-economic factors, are other limitations of this study.

## Conclusion

 The occurrence of animal bites in Babol over 8 years showed an increasing trend, indicating growing concern and the need for effective preventive measures. Based on the characteristics under investigation and the examination of bite trends and patterns, these results can provide valuable insights for public health authorities, policymakers, and healthcare providers to formulate intervention strategies, awareness campaigns, and preventive actions to address specific patterns and trends in animal bites. Therefore, given the importance of this disease and its financial burdens, it is recommended to use prevention methods to control stray dogs, vaccinate domestic dogs, and increase public awareness.
